# In Vitro Detection of Lactate and Uric Acid Based on Adaptive Graphene Oxide Membranes

**DOI:** 10.1002/smsc.202300264

**Published:** 2024-02-27

**Authors:** Bo Fang, Zeyu Zhao, Jie Ma, Heng Li, Feng Yan, Xiaoming Tao

**Affiliations:** ^1^ Research Institute for Intelligent Wearable Systems The Hong Kong Polytechnic University Hong Kong 999077 China; ^2^ School of Fashion and Textiles The Hong Kong Polytechnic University Hong Kong 999077 China; ^3^ Department of Applied Physics The Hong Kong Polytechnic University Hong Kong 999077 China; ^4^ Department of Building and Real Estate The Hong Kong Polytechnic University Hong Kong 999077 China

**Keywords:** biomarker, graphene oxide membrane, molecular detection, non‐invasive test, organic electrochemical transistor

## Abstract

In vitro detection of small molecules in the human circulatory system contributes a lot to prompt personalized healthcare and early disease diagnose. The precise detection requires to select target molecules for real‐time noninvasive identifications, which remains a challenge derived from the lack of both molecule‐sieving techniques and rapid detection tools. Herein, positively charged polymer chains into graphene oxide membranes are seeded to tune their interlayer spacing precisely and persistently within a range of 8.67–13.75 Å. The adaptive yet stable nanocapillaries allow membranes to sieve small molecules with fitted sizes, for example, salts and biomarkers. The molecule‐sieving membranes with organic electrochemical transistors, further achieving an in vitro detection of lactate and uric acid with a high accuracy, are coupled. Based on this finding, a wearable sweat lactate rapid test kit, which detects sweat lactate secreted from human body within a few minutes, is demonstrated.

## Graphical abstract

An in vitro biomarker monitoring system integrating molecule‐sieving graphene oxide membranes and planar organic electrochemical transistors realizes the quantitative detection of sweat lactate and uric acid. Building on this principle, a wearable sweat biomarker rapid test kit is developed to detect lactate from human body with a few minutes.

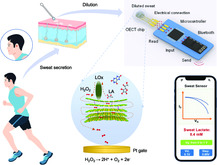

## 1 Introduction

Molecule detection techniques monitor the transport behaviors and quantities of molecules at nanometer and subnanometer scale, which have demonstrated great potentials in frontier domains including bioanalytic separation,^[^ ^1^, ^2^ ^]^ green energy harvest,^[^ ^3^, ^4^ ^]^ and nuclear water management.^[^ ^5^, ^6^ ^]^ Among them, in vitro detection of small molecules, such as lactate and uric acid, in body fluids is quite helpful for noninvasive monitoring and wearable healthcare. Specifically, noninvasive detection of lactate is highly important for respiratory and exercise condition prediction, while the real‐time detection of uric acid plays a significant role for monitoring circulatory system and hemolytic uremia. To do this, molecule‐sieving technology is on the first demand to precisely select target molecules, followed by coupling with reliable detection tools. Benefiting from low energy cost, large production scale, and high work efficiency, membrane technology has stood out in recent research to handle scientific and engineering issues during the molecule‐sieving process.^[^ ^7^, ^8^, ^9^, ^10^, ^11^ ^]^ Suitable membranes should have nanopores to block or allow the pass of molecules. Building on this principle, membranes with two mainstream kinds of nanopores have been proposed, that is, fixed and adaptive. Fixed membranes with constant pore size sieve specific molecules with fitted diameters, generally requiring elaborate predesign. For example, polymerizing stiff bridged bicyclic rings into membranes achieved a high surface area (>1000 m^2^ g^−1^) for separating H_2_ and O_2_, and metal–organic framework‐aggregated membranes with nanometer‐sized channels were able to remove CO_2_ and H_2_S from natural gas.^[^ ^12^, ^13^ ^]^ However, adaptive membranes have received tremendous attention due to easy preparation and adjustable pore size.^[^ ^14^, ^15^, ^16^ ^]^

Graphene oxide (GO) membrane is a typical adaptive sieve possessing adjustable nanopores with the evolution of surroundings, especially in solvents.^[^ ^17^, ^18^, ^19^, ^20^ ^]^ Hydrating GO membranes in water immediately induce the intercalation of a few layers of H_2_O molecules between GO sheets, which forms the uniform network of nanocapillaries with interlayer spacing (*d*) increasing from pristine <8 Å to saturated >13 Å.^[^ ^16^ ^]^ Such nanocapillaries work as molecular sieves to block all solutes with larger hydrated diameters while taking in smaller solutes. Unfortunately, the free swelling of GO membranes in water has generally caused a relatively large yet nonadjustable permeation cutoff of 13–14 Å.^[^ ^18^ ^]^ Previous efforts, such as partial reduction,^[^ ^21^ ^]^ physical confinement,^[^ ^16^ ^]^ and cationic control,^[^ ^22^ ^]^ have been proposed to manage the swelling process and tune the permeation cutoff. So far, most solutions have still been limited by either the low production efficiency or the narrow range of *d* regulation. It is challenging yet desired to find a feasible method to precisely control *d* of GO membranes at a wide range.

In this work, we proposed a charge‐compensated GO membrane to precisely sieve biomarkers, further achieving their rapid detection by coupling with planar organic electrochemical transistors (OECTs). We doped positively charged polyethyleneimine (PEI) chains between negatively charged GO sheets. With the changing contents of PEI, *d* of swollen GO membranes was precisely tuned from 8.67 to 13.75 Å through the electrostatic interactions between PEI and GO. Furthermore, the interlinked membranes showed reliable durability of both structures and performance. At smaller *d*, the charge‐compensated membranes excluded small molecules. At larger *d*, the transportation of biomarkers was blocked, while the entry of hydrogen peroxide was unimpeded. We assembled the molecule‐sieving membranes with OECT devices and realized the accurate in vitro detection of biomarkers, for example, lactate and uric acid, due to the selective pass of biomarkers and hydrogen peroxide. Finally, we demonstrated a user‐friendly sweat lactate rapid test kit, by which people could conduct real‐time monitoring of lactate secreted from body fluids. Our work provides a convenient solution to regulate *d* of GO membranes. The successful coupling of molecule‐sieving membrane and OECT devices also suggests the great potential of this technology in fields covering noninvasive detection, smart healthcare, and wearable medical system.

## 2 Results and Discussion

**Figure 1:**
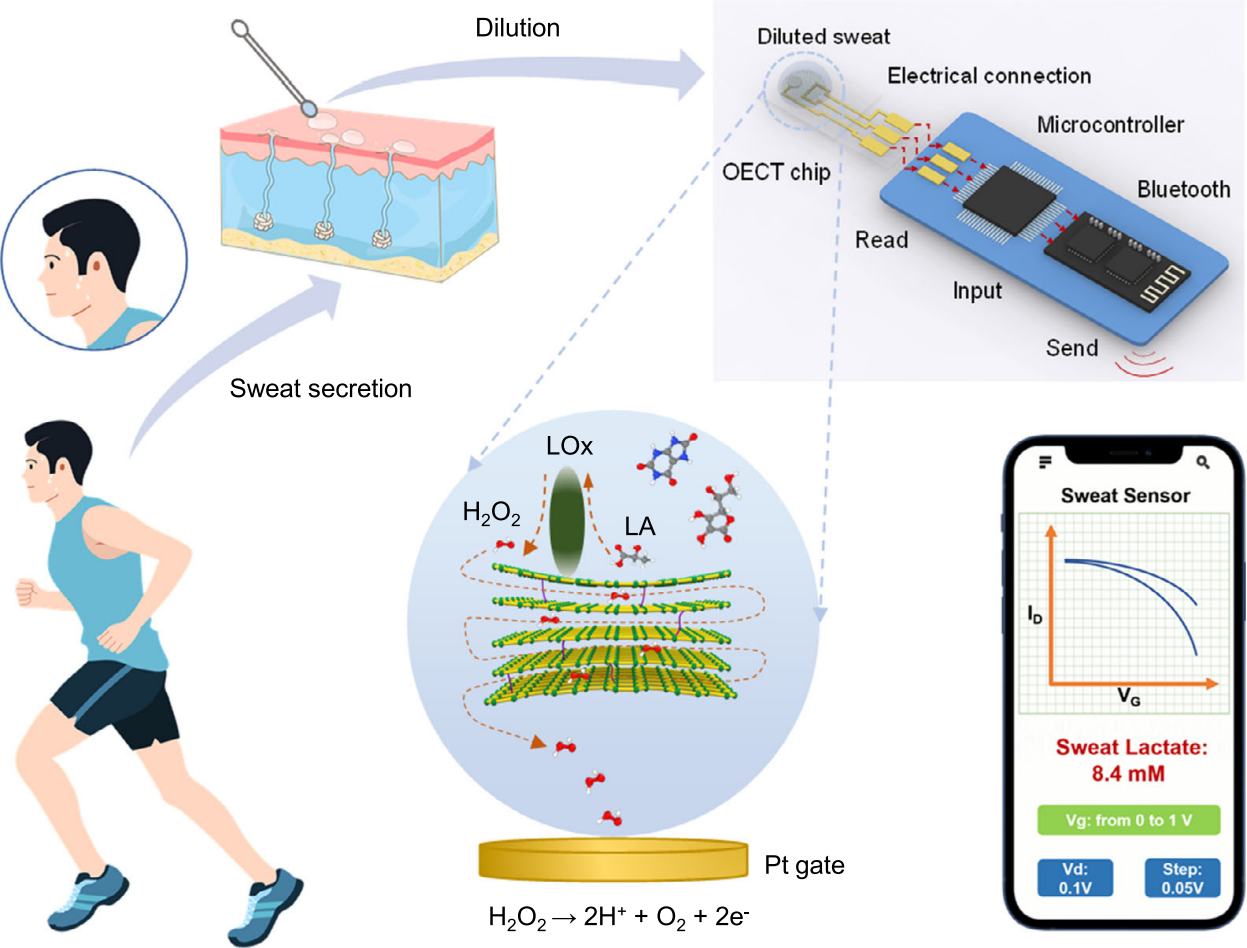
Rapid sweat lactate detection based on adaptive GO membranes. Sweat samples secreted from human body will be diluted and added on an OECT chip, which is modified by an adaptive GO membrane and lactate oxidase (LO*x*). Among the biomarkers in sweat, lactate (LA) is selectively oxidized into H_2_O_2_ by LO*x*. The produced H_2_O_2_ passes through the nanocapillaries of adaptive GO membrane due to the small size, while the pass of biomarkers with large size will be blocked. H_2_O_2_ immediately transfers two electrons to Pt gate electrode, further sending the signal of LA concentration to the mobile phone through the processing of microcontroller and Bluetooth within 3 min.

We achieved the in vitro molecular detection by coupling adaptive GO membranes with a well‐designed OECT chip. Dominated by a physical confining process, this strategy is superior to previously reported chemical interaction‐based GO biosensors due to the low cost, high durability, and easy operation. To illustrate this, we explained the rapid test of sweat lactate in **Figure** **1**. The sweat sample containing numerous autocrine charged biomarkers, such as lactate (LA), ascorbic acid (AA), dopamine (DP), and uric acid (UA), was extracted and diluted with a fixed proportion, further doping on an OECT chip modified by an adaptive GO membrane. Lactate oxidase (LO*x*) rooted on the membrane selectively oxidized LA into hydrogen peroxide (H_2_O_2_), immediately transferring two electrons to the Pt gate electrode of OECT chip. The electron transfer would generate the quantitative shift of electrical response of OECT, which was written by a microcontroller and received by a mobile phone via Bluetooth. In principle, through the calculation to the shift amplitude by the software package of the mobile device, the exact concentration of LA in sweat sample would be read and displayed. The main concern comes from the direct contact of charged biomarkers to gate electrode, which would deflect the quantitative relationship between LA concentration and electrical response. To address this concern, we inserted an adaptive GO membrane with adjustable nanopores on OECT electrodes, which blocked biomarkers and released H_2_O_2_.

We fabricated the adaptive GO membranes (≈19.6 cm^2^) by vacuum filtration. Aqueous dispersions of GO sheets with an average size of 2–3 μm (Figure S1, Supporting Information) were mixed with PEI dispersions at different mass ratios. Note that pristine GO sheets are negatively charged,^[^ ^23^ ^]^ and we controlled the mass fractions of positively charged PEI with <5% at a low temperature (<20 °C) to avoid rapid aggregation and possible reduction, for example, 1.23% (GO‐PEI‐1), 2.44% (GO‐PEI‐2), 3.57% (GO‐PEI‐3), and 4.76% (GO‐PEI‐4). The built interactions between PEI‐GO sheets were also evidenced by the emerging N–O interaction signals in the Fourier‐transform infrared (FTIR) spectrum of GO‐PEI membranes (Figure S2, Supporting Information). The obtained GO and GO‐PEI membranes have uniform microstructures with a thickness of ≈43 nm.

**Figure 2:**
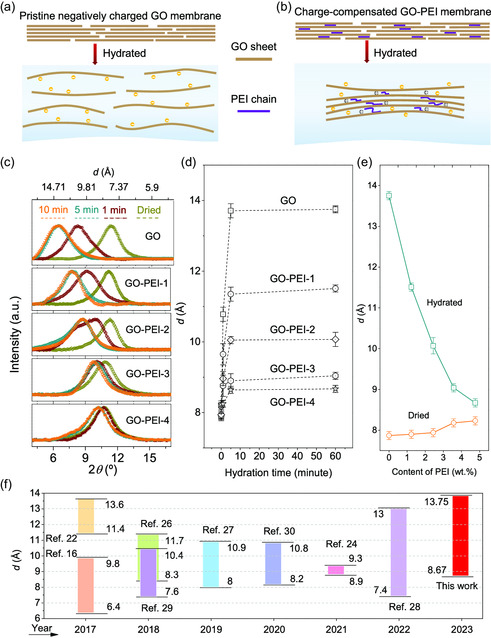
Tuning the interlayer spacing of membranes. Schematic showing the evolution of hydrated a) GO and b) GO‐PEI membranes. c) XRD spectra of membranes during the 10‐minutes hydration process. In all the five samples, dark yellow curves collected at dried state, wine curves collected at the 1st minute, dark cyan curves collected at the 5th minute, and orange curves collected at the 10th minute. d) The relationship between *d* and hydration time. e) The relationship between *d* and the content of PEI. f) Recently proposed adaptive GO membranes for achieving different *d*.

The enhanced crosslinking network induced by doped PEI chains tuned the interlayer spacing of hydrated GO‐PEI membranes precisely. To understand the antiswelling roles of PEI chains, we directly observed the microstructures of hydrated membranes by high‐resolution transmission electron microscopy (TEM) after freeze drying. For the pristine GO membranes, the thickness increased from ≈43 to ≈83 nm after undergoing sufficient swelling (Figure S3, Supporting Information). Although it is difficult to clearly recognize the stacking status of distorted GO layers due to their poor degree of crystallinity, we still determined *d* (≈1.3 nm) of swollen GO membranes at the regular region (Figure S3b, Supporting Information). Based on the sharply increased *d* and distorted patterns in TEM observations, we tried to explain the evolution of GO molecules during the hydration process. As illustrated in **Figure** **2**a, GO layers drifted away from each other with the insertion of H_2_O molecules. This process is in principle uncontrollable due to the mutual repulsion of negatively charged GO sheets,^[^ ^24^ ^]^ which could be addressed by the charge compensation of positively charged PEI chains. For example, the thickness of the hydrated GO‐PEI‐3 membranes is decreased to ≈57 nm (Figure S3c, Supporting Information). Interestingly, with the suppression of the swelling process, the arrangement of GO layers appeared more regular with a decreased *d* of ≈0.9 nm (Figure S3d and S4, Supporting Information). We speculate that the controlled swelling is ascribed to the charge‐compensated action of PEI, which contained the motion of GO layers by the electrostatic interactions (Figure 2b).

We tracked *d* of membranes using X‐ray diffraction (XRD) during the hydration process. As shown in Figure 2c, at the dried state, the sharp XRD peaks (dark yellow curves) of the prepared GO and GO‐PEI membranes suggest their well‐ordered structures.^[^ ^25^ ^]^ Further, the closed peaks at 10.7–11.2 2*θ* degree proved that the doping of PEI chains caused negligible influences on the GO stacking at the dried state. After being hydrated in water, XRD peaks became wide and showed a blueshift until ≈10 min (orange curves), reflecting that H_2_O molecules weakened the interlayer interactions and enlarged the interlayer spacings. This finding accords well with our TEM observations and interpretations in Figure S3 and S4, Supporting Information. During 10 min of hydration, the gradually decreased shift of XRD peaks from GO to GO‐PEI‐4 clearly demonstrated the effective suppression of GO layers’ free swelling by PEI chains doping. Based on XRD results, we calculated *d* of membranes by Bragg's law. As summarized in Figure 2d,e, pristine GO membranes had a *d* of ≈7.87 Å. which slightly increased to ≈8.23 Å in the case of GO‐PEI‐4 membranes. In water, *d* increased to the largest values in 5 min. The roughly unchanged *d* from 5 to 10 min shows the reliable structural stability of GO‐PEI membranes for durable operation. The structural durability of swollen GO‐PEI membranes could also be demonstrated by our ultrasonic tests. As shown in Figure S5 and Movie S1, Supporting Information, the pristine GO membrane disintegrated in 30 s ultrasonic test, while GO‐PEI‐4 membrane maintained the structural integrity. Specifically, after sufficient dehydration, *d* of GO membranes saturated at ≈13.75 Å, approaching that of the hydrated GO membranes (≈13.5 Å) in previous works.^[^ ^16^, ^17^, ^25^ ^]^ The introduction of PEI chains precisely tuned *d* to decreasing saturated values, for example, ≈11.51 Å for GO‐PEI‐1, ≈10.07 Å for GO‐PEI‐2, ≈9.04 Å for GO‐PEI‐3, and ≈8.67 Å for GO‐PEI‐4, respectively. As shown in Figure 2f, the wide range of *d* between 8.67 and 13.75 Å makes the adaptive GO membranes superior to that fabricated by recently proposed techniques, such as pressure confinement,^[^ ^16^, ^26^ ^]^ cationic control,^[^ ^22^ ^]^ nanoparticle/polymer filling,^[^ ^24^, ^27^, ^28^ ^]^ and crosslinking.^[^ ^29^, ^30^ ^]^

**Figure 3:**
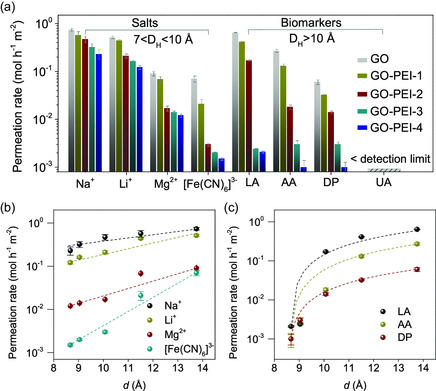
Permeation tests of small molecules through membranes. a) Permeation rates of salts and biomarkers through all the membranes. The relationship between permeation rates and *d* when b) feeding salts and c) biomarkers, respectively.

The well‐controlled *d* and structural stability allowed GO‐PEI membranes to sieve the transport of small molecules. We built two semiclosed glass containers to study the sieving performance of membranes, as shown in Figure S6, Supporting Information, similar to ones previously reported.^[^ ^18^, ^31^ ^]^ All the membranes were hydrated in water for 1 h before being subject to permeation experiments. Aqueous dispersions containing 1 M common salts, for example, NaCl, LiCl, MgCl_2_, K_3_[Fe(CN)_6_], were respectively added. **Figure** **3**a summarizes the permeation rates of ions through different membranes (Figure S7, Supporting Information). The permeation of all the ions declined with the increasing contention of PEI in membranes due to the gradually narrow nanochannels. In the case of ions with smaller hydration diameter (*D*_H_), such as Na^+^ (≈7.16 Å) and Li^+^ (≈7.6 Å), the declines were relatively slight within one order of magnitude, while the declines were distinct when the ion sizes were larger, for sample, over one order of magnitude for Mg^2+^ (≈8.56 Å) and even over two orders of magnitude for [Fe(CN)_6_]^3−^ (≈9.5 Å). The exponential dependence of ion permeation rates on *d* is consistent with the previously proposed dehydration‐limited mode (Figure 3b).^[^ ^16^ ^]^ This suggests that the penetration of ions through GO‐PEI membranes proceeds with conquering energy barriers by the removal of hydration shell, wherein ions with strong and large shells would hardly pass. As a result, membranes with smaller *d* tended to severely restrict the transport of ions with larger *D*_H_.

Therefore, the size‐based selectivity was much obvious in the cases of biomarkers with large volumes. We conducted permeation experiments to biomarkers frequently found in body fluids (Figure S8, Supporting Information), for example, LA, AA, DA, and UA. *D*_H_ of biomarkers is generally beyond 10 Å. As shown in Figure 3a, the permeation rates of all the biomarkers decreased by over two orders of magnitude from GO to GO‐PEI‐4 membranes, and the permeation rate of UA was even lower than our detection limit. The transport behaviors of biomarkers are not the same as that of salts. When fitting the permeation rates of biomarkers with *d*, as shown in Figure 3c, we found that the permeation of biomarkers showed a sharp speed reduction at a small *d* of <11 Å. This implies that, besides the dehydration shell removal process, there are other associated factors retarding the pass of biomarkers. We assumed that the strong hydrogen bonding formed between GO sheets and the rich oxygen‐containing groups of biomarkers could be an additional factor dragging the moving molecules, which is inexistent in salts‐related system. The strong dependence of permeation rates on *d* makes GO‐PEI membranes to sieve biomarkers with very high selectivity. For example, GO‐PEI‐3 and GO‐PEI‐4 membranes with *d* of <10 Å excluded all the tested biomarkers with a permeation rate of <0.01 mol^−1^ h^−1^ m^2^. In body fluids, H_2_O_2_ is an important intermediate molecule generating from the metabolism of biomarkers at energy‐demanding circumstance. We also studied the transport of H_2_O_2_ through molecule‐sieving membranes. Interestingly, during our 30‐day cycle test, H_2_O_2_ showed almost unimpeded transport from GO to GO‐PEI‐3 membranes, while a retention of ≈37% was recorded when passing though GO‐PEI‐4 membranes (Figure S9, Supporting Information). This result revealed a cutoff of <9.04 Å for the permeation of H_2_O_2_ in aqueous solutions. The almost unchanged microstructures of GO‐PEI‐3 membranes from beginning to end (SEM images in Figure S9, Supporting Information) further demonstrated their reliable operation stability.

**Figure 4:**
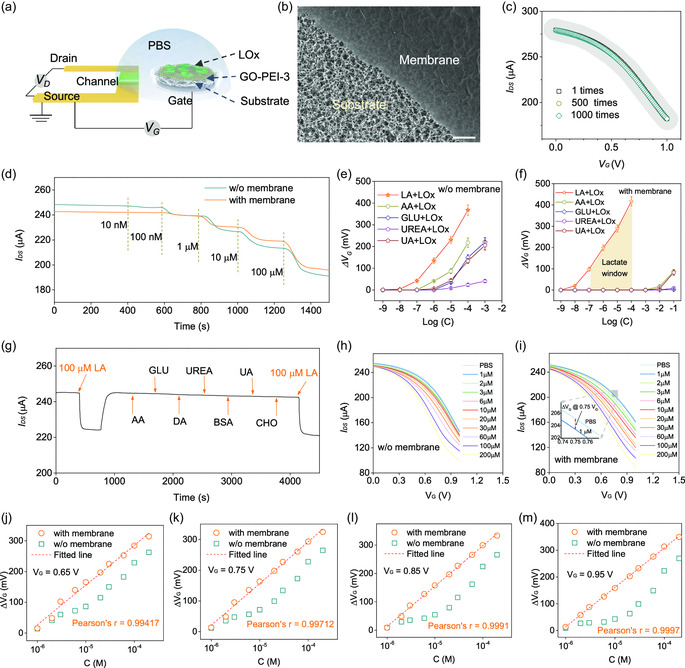
Structure and performance of modified OECT by GO‐PEI‐3 membranes. a) Schematic illustrating the structure of modified OECT. b) Microstructures of GO‐PEI‐3 membrane supported on a porous polymer substrate. c) Transfer curves of modified OECT during 3000 times test. d) Sensitivity test to lactate on bare gate electrodes and on modified gate electrodes. e,f) Relationship between gate voltage shift (Δ*V*_G_) and logarithmic concentration of biomarkers (log (C)) with the feed of lactate oxidase (LO*x*). g) The response and durability test of modified OECT to lactate at a feed of 100 μM. No response was monitored with the successive addition of AA, glucose (GLU), DA, urea, BSA, UA, and cholesterol (CHO). Transfer curves of OECT h) without and i) with the coating of GO‐PEI‐3 membrane. The fitted Δ*V*_G_–log (C) curves at the gate voltage of j) 0.65 V, k) 0.75 V, l) 0.85 V, and m) 0.95 V, respectively. Scale bar in b: 10 μm.

Based on the selective pass of biomarkers and H_2_O_2_, we used GO‐PEI‐3 membranes to conduct in vitro tests of biomarkers by coupling with OECT. The real‐time monitoring of biomarkers in the human circulatory system is helpful for prompt personalized healthcare during intense exercise, as well as for early‐disease diagnosis in daily life.^[^ ^32^, ^33^, ^34^, ^35^ ^]^ OECT is a high‐performance three‐electrode transducer working in electrolyte medium.^[^ ^36^, ^37^, ^38^ ^]^ Doping the oxidase of specific biomarkers on OECT can, in principle, achieve their highly selective detection by the electron exchange between oxidase‐induced H_2_O_2_ and gate electrode.^[^ ^39^, ^40^ ^]^ Unfortunately, in practical circumstance, the direct contact between biomarkers and gate electrodes will distort the electrical response by electrostatic interactions and electric dipole.^[^ ^41^, ^42^ ^]^ We modified the three‐terminal OECT devices by planting a GO‐PEI‐3 membrane on gate electrode to avoid the invading of biomarkers. As illustrated in **Figure** **4**a and S10, Supporting Information, a planar OECT device with a thickness of ≈100 nm was in service, where an oxidation‐resisting Cr/Au composite served as the drain/source electrodes, a highly sensitive Ti/Pt composite worked as the gate electrode, and poly(3,4‐ethylenedioxythiophene)‐poly(styrenesulfonate) (PEDOT:PSS) acted was the semiconducting channel. Oxidase of lactate (LO*x*) was rooted on GO‐PEI‐3 membrane, which was supported by a porous and electroneutral polymer substrate (Figure 4b and S11, Supporting Information). The insertion of molecule‐sieving membranes does not influence the operation stability of OECT, as demonstrated by the unchanged transfer curves during 1000 cycle tests (Figure 4c). The unimpeded pass of H_2_O_2_ through the membrane and microporous substrate enables the modified gate to detect lactate at the similar range to that of the bare electrode. As plotted in Figure 4d, both electrodes responded to lactate at the concentrations from 10 nM to 100 μM.

The selective pass of lactate and H_2_O_2_ through GO‐PEI‐3 membrane rectified the electrical response of OECT. Under the directional catalysis of LO*x*, lactate produces metastable H_2_O_2_, which immediately transfers two electrons to gate electrode, further resulting in the shift of effective gate voltage (Δ*V*_G_). In principle, Δ*V*_G_ has linear dependence on the logarithmic value of lactate concentration (log(C)), being expressed as follows.

(1) $$\Delta V_{G}=2.30\left(1+\frac{C_{E-C}}{C_{G-E}}\right)\frac{kT}{2q}\log\left(C\right)+A$$

where *k* is Boltzmann constant, *T* is ambient temperature, *q* is electron charge, *A* is constant, and *C*_E–C_ and *C*_G–E_ are the capacitances of the two electric double layers close to the channel and the gate, respectively.^[^ ^41^, ^42^, ^43^, ^44^, ^45^ ^]^ The linear dependence of Δ*V*_G_ on log(C) represents the electrical response of OECT to lactate, which makes OECT a powerful tool to detect biomarkers in a noninvasive manner. On bare electrodes, as shown in Figure 4e, the electrical response to lactate was interfered by the contact of other biomarkers, for example, AA, glucose (GLU), urea, and UA, at a very low detection limit (≈10^−7^ M). With the sieving of GO‐PEI‐3 membrane, the interference from other biomarkers was precluded, indicated by their much‐improved detection limit of 10^−3^ M, roughly an improvement of four orders of magnitude (Figure 4f), while the detection limit of lactate remained at ≈10^−9^ M. Further, we found a lactate window at the concentrations between 10^−7^ and 10^−4^ M, wherein, as shown in Figure 4f, Δ*V*_G_ showed clear linear dependence on log(C) with no detectable noise. In the lactate window, the response of modified OECT to lactate is highly specific. During our 4500 s test, as shown in Figure 4g, the drain–source current (*I*_DS_) occurred an immediate decline recovery with the addition removal of lactate, while the addition of other biomarkers did not generate any response (see the typical control experiments in Figure S12–S14, Supporting Information). We also collected output curves of OECT devices by doping lactate at a concentration from 1 to 200 μM (Figure 4h,i). The results were summarized in Figure 4j–m. We found that, at various gate voltages (*V*_G_) from 0.65 to 0.95 V, Δ*V*_G_ of all the curves collected on modified electrodes shows linear dependence on log(C) with Pearson's r beyond 0.99 (orange curves), while the dependence of Δ*V*_G_ on log(C) is irregular on bare electrodes (dark cyan curves).

**Figure 5:**
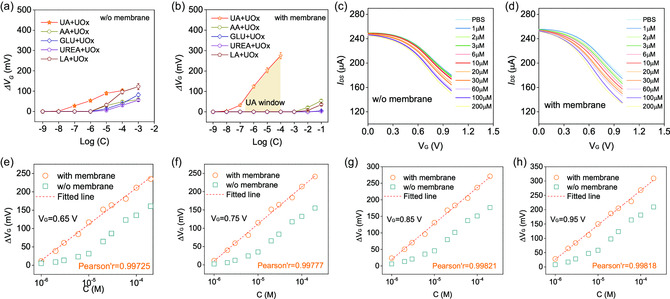
Uric acid detection of modified OECT. a,b) Relationship between Δ*V*_G_ and log (C with the feed of uric oxidase (UO*x*). Transfer curves of OECT c) without and d) with the coating of GO‐PEI‐3 membrane. The fit Δ*V*_G_–log (C) curves at the gate voltage of e) 0.65 V, f) 0.75 V, g) 0.85 V, and h) 0.95 V, respectively.

Besides lactate, we also demonstrated the improved selectivity of modified OECT to UA by rooting UA oxidase (UOx). As shown in **Figure** **5**a,b, the use of GO‐PEI‐3 membrane improved the detection limits of other biomarkers from ≈10^−6^ to ≈10^−3^ M, and the detection limit of UA was not affected (≈10^−8^ M). We also recorded a UA window at the concentration from ≈10^−6^ to ≈10^−4^ M, wherein Δ*V*_G_ is linearly dependent on increasing log(C). Based on the collected output curves from 1 to 200 μM in Figure 5c,d, we obtained the linear relationship between Δ*V*_G_ and log(C) with Pearson's *r* beyond 0.99 on modified electrodes (orange curves in Figure 5e–h). The ruleless response of Δ*V*_G_ to log(C) on bare electrodes (dark cyan curves in Figure 5e–h) originated from the uncontrolled electrical contact between UA molecules and gate electrode.

**Figure 6:**
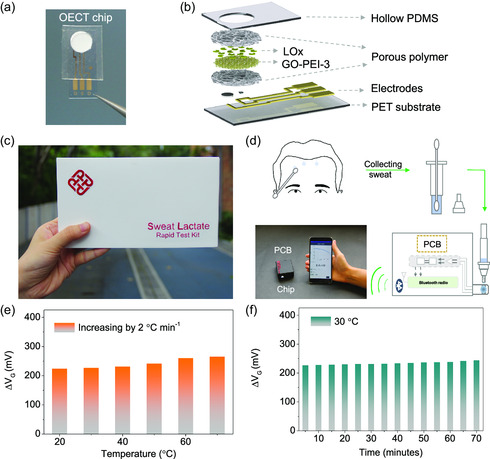
Structure and operation flow of sweat lactate rapid test kit. a) Optical picture and b) schematic of OECT chip modified by GO‐PEI‐3 membrane. c) Optical picture and d) operation flow of sweat lactate rapid test kit. Adding the diluted sweat on modified gate electrode, reading the analyzing the electrical signals by a microcontroller, and then sending the results to mobile devices via Bluetooth. e) The influence of surrounding temperatures on kit. The temperature rises at 10 °C per 5 min. f) Stability test of kit at 10 °C.

With the rectified OECT device by GO‐PEI‐3 membrane, we demonstrated a sweat lactate rapid test kit to detect sweat lactate within a few minutes, promoting previous conceptional demonstrations to practical applications. We fabricated a portable OECT chip (**Figure** **6**a). As illustrated in Figure 6b, the multilayered OECT chip consists of six layers, that is, a hollow PDMS on the top layer to allow the handy addition of sweat samples as well as protecting the device, a GO‐PEI‐3 membrane in the middle layer sandwiched by two porous polymer substrates to sieve biomolecules, LO*x* rooted on the GO‐PEI‐3 membrane, and metal electrodes grown on a PET board, which supported the whole chip. To build the sweat lactate rapid test kit, we connected the OECT chip with a microcontroller and Bluetooth. As shown in Figure S15, Supporting Information, after adding sweat samples on the OECT chip, the concentration signal of lactate will be rapidly analyzed and read by a microcontroller, being further sent to mobile devices through Bluetooth. The practical kit is a handy 3D‐printed box containing several units (Figure 6c and S16, Supporting Information), that is, swabs for collecting sweat samples, commercial reagent bottles (loading 5 mL phosphate‐buffered saline (PBS)) for diluting sweat samples, OECT chips, and a printed circuit board (PCB) printed with signal processing modules. Figure 6d explains the operation flow (see Movie S2, Supporting Information, for details). Considering the temperature‐induced effects on the reaction efficiency of H_2_O_2_, we studied the influences of surrounding temperatures to the kit performance. As shown in Figure 6e,f and S17, Supporting Information, the kit guaranteed reliable operation states at near room temperatures, at least 20–40 °C. We recruited human participants to monitor sweat lactate during the continuous exercise of badminton and running. As shown in Figure S18, Supporting Information, the kit worked well to conduct real‐time detection of sweat lactate, helpful for the prediction of human fatigue during sports exercise. Commercial sweat detection approaches generally operate based on spectrum technologies, such as Lambert–Beer's law, midinfrared spectrum shift, and surface‐enhanced Raman scattering, which is semi‐/nonquantitative. The sweat rapid test kit demonstrated a reliable quantitative detection tool for daily use.

## 3 Conclusion

Over the past decade, we have witnessed great efforts in seeking adaptive and stable membranes with submicrometric pores to efficiently sieve guest molecules. However, the progress has been limited by either unsatisfying production efficiency or insufficient regulation range of pore sizes. We introduce a feasible charge‐compensation method to tune *d* of GO membranes during hydration. With the superb structural stability, the well‐controlled *d* makes GO‐PEI membranes useful for the in vitro sieve of small molecules with suited hydrated sizes, such as salts and biomarkers. Importantly, the selective pass of biomarkers and H_2_O_2_ through GO‐PEI membranes contributes to the correction of OECT response, leading to the rapid detection of biomarkers. We demonstrated a user‐friendly sweat lactate rapid test kit to monitor lactate within 3 min, which was helpful for the instantaneous determination of human fatigue and hyperlactatemia‐related illness. Besides the rapid detection technology, the scalable molecule‐sieving membrane can further be applied in frontier fields such as hemodialysis system, smart medical care, and ion‐based devices.

## 4 Experimental Section

4.1

4.1.1

##### Materials

Aqueous solutions of GO with an average flake size of 2–3 μm were purchased from Gaoxitech. PEI was purchased from Aladdin. PEDOT:PSS (Clevios PH 500) was obtained from Heraeus Ltd. Zinc acetate, dimethyl sulfoxide, PDMS, glycerin, nafion aqueous solution (5%), bovine serum albumin (BSA), lactate oxidase (LO*x*), lactic acid (LA), glucose (GLU), dopamine (DA), and cholesterol (CHO) were bought from Sigma‐Aldrich, Inc. Urea, urea acid (UA), urate oxidase (UOx), ascorbic acid (AA) were all provided by J&K Scientific (Hong Kong) Ltd. PBS was bought from Thermo Fisher Scientific Inc. AZ5214 and SU‐82002 photoresists were purchased from MicroChem Corp.

##### Characterization

Atomic force microscope images were obtained by Scanning Probe Microscope (Bruker MultiMode 8). FTIR spectra were recorded by FTIR (Bruker, VERTEX 70, HYPERION 2000). TEM pictures were recorded by Spherical Aberration TEM (JEOL‐2010). Before TEM observations, all the membranes were hydrated in deionized water for 1 h and then freeze dried. XRD spectra were collected from the Rigaku SmartLab system equipped with a 9 kW rotating‐anode X‐ray source (*λ* ≈ 1.54 Å) and a high‐quality semiconductor detector. SEM pictures were recorded on FESEM (Tescan MAIA3). The transfer curves of OECTs were recorded by sweeping gate voltage (*V*_G_) from 0 to 1 V with the fixed drain–source voltage (*V*_DS_) at 0.1 V, and the relative change of the gate voltage Δ*V*_G_ was calculated. The real‐time channel current response (I–T curve) of OECTs to the addition of various biomarkers was recorded with Keithley source meters (Keithley 2400). *V*_DS_ was fixed at 0.1 V, and *V*_G_ was fixed at 0.5 V during the I–T test.

##### Characterization: Fabrication of Membranes

Before being mixed, the concentrations of GO and PEI were diluted to below 0.5%. In a cold bath (≈15 °C), PEI solutions were slowly added into GO dispersions drop by drop until the target mass ratios were achieved. The prepared GO‐PEI solutions were deposited into thin membranes after being subject to vacuum filtration. The prepared films dried at ambient for 24 h before next processing.

##### Characterization: Filtration Tests Through Membranes

All the salts used in our tests, including NaCl, LiCl, MgCl_2_, K_3_[Fe(CN)_6_], were prepared in deionized water at the same concentration of 1 M in advance. Due to the limited saturation solubility, the concentrations of biomarkers were different, to be specific, lactate for 1 M, ascorbic acid for 0.5 M, dopamine for 0.1 M, and uric acid for 0.4 mM. The concentration of H_2_O_2_ was also controlled at 1 M. A standard H‐mode electrolytic tank with a capacity of 100–100 mL and a channel diameter of 16 mm was for prepared for filtration tests. Before testing, all the membranes were hydrated in pure water for the removal of possible impurities and sufficient swelling of GO layers. The hydrated membranes were locked in the channel and supported by two waterproof rubber pads. The solutions of salts, biomarkers, and H_2_O_2_ were injected to the feed side, while the permeation side was filled with deionized water at the same volumes (≈100 mL). Then, the ion or biomarker concentrations in the permeation side were recorded by an ion conductance meter in every other hour.

##### Characterization: Fabrication of OECT Devices

The OECT device was supported on the PET substrates and prepared by metal layers sputtering, semiconductors’ spin coating, and photolithography‐encapsulation technology. In detail, AZ5214 photoresist was spin coated on the PET substrates and transferred the predesigned pattern using Karl Suss MA6 Mask aligner. Cr/Au (thickness: ≈10/≈100 nm) layers and Ti/Pt (thickness: ≈10/≈90 nm) layers, serving as source/drain electrodes and gate electrodes, respectively, were then successively deposited on the PET substrates by radio frequency magnetron sputtering system. After two times deposition, the whole device was encapsulated by SU‐8 photoresist to protect the metal electrodes from the aqueous electrolyte. During this procedure, semiconducting channel and gate electrodes were exposed without encapsulation. The channel window was opened with a similar photolithography process, further spin coated with aqueous solutions of PEDOT:PSS, which consisted of 90% Clevios PH‐500, 5% DMSO, and 5% glycerin, and then annealed at 110 °C in nitrogen for 20 min. The residual PEDOT:PSS was removed by acetone to expose a channel with a width of 120 μm and a length of 30 μm, respectively. In the case of modified OECT by GO‐PEI‐3 membrane, Pt gate electrodes of the cleaned OECTs were first modified with a little Nafion solution (5 mg mL^−1^) and GO membrane with PDMS shield was then covered on the gate electrodes. The LO*x* solution (200 U per gate electrode) was coated on the surface of the GO membrane and immobilized by chitosan acetic solution (8 mg per gate electrode). After each step of the solution modification, the device was dried at ambient with a constant temperature of 4 °C. Before sensing measurements, the prepared device was rinsed thoroughly with PBS solution to remove the unanchored residues.

##### Characterization: Operation Flow of OECT Chip

Commercial Ryan swabs (63 μL capacity) collected sweat secreted from body until saturation. The saturated swabs were diluted in reagent bottles containing 5 mL PBS with a dilution ratio 0.016:1 by stirring and squeezing. In this way, the possible concentrations of sweat lactate in normal human body (1–100 mM) were diluted into the lactate window of our modified OECT devices (≈10^−4^ to 10^−1^ mM). One drop of PBS dilutions was added on the OECT chip. The generated output signals were transmitted to the microcontroller on the PCB, which read the accurate value of lactate concentrations by their linear relationship and sent the results to a loaded software through Bluetooth.

##### Characterization: Real‐Time Test to Human Participants by the Sweat Lactate Rapid Test Kit

Two healthy male participants were recruited for the tests based on the program approved by Human Subjects Ethics Application Review System of the Hong Kong Polytechnic University (no. HSEARS20220527001). The health state of participants was continuously monitored by physiological signals, including calorie consumption, blood oxygen, and heart rate, as introduced in the main text. During the badminton exercise, the sweat samples were collected by swabs loaded in the sweat lactate rapid test kit at every 10 min. The other physiological signals were also recorded at the same time. Specifically, calorie consumption and heart rate were recorded by a commercial sports bracelet, blood oxygen was measured by a medical oximeter, and blood lactate was collected by a commercial blood lactate meter. The collection of sweat samples during running was conducted at every 1000 m distance. The running speed was controlled between 4 and 5 min per kilometer. The collected samples were diluted into PBS loaded in the reagent bottles, and then a drop of diluted samples was added on the OECT chip for the following tests.

## Conflict of Interest

The authors declare no conflict of interest.

## Author Contributions

X.T., F.Y., H.L., and B.F. supervised this study. B.F. designed and conducted the main characterizations. Z.Z. fabricated and characterized the transistors. B.F. and J.M. fabricated the membranes and collected the sweat samples. B.F., Z.Z., and J.M. conducted the permeation tests and designed the sweat lactate rapid test kit. B.F. wrote the manuscript.

## Supporting information

Supplementary Material

## Data Availability

The data that support the findings of this study are available in the supplementary material of this article.
