# Supplementary material for: In Vitro Detection of Lactate and Uric Acid Based on Adaptive Graphene Oxide Membranes
Source: Small Sci. 2024 Feb 27;4(4):2300264. doi: 10.1002/smsc.202300264 (PMC11935139; doi:10.1002/smsc.202300264)
Supplement: Supplementary file 1 — Supplementary Material [file SMSC-4-2300264-s001.zip › smsc.202300264-sup-0001-suppdata-S1.pdf]

Supplementary Information for

In Vitro Molecular Detection of Lactate and Uric Acid Based on Adaptive Graphene Oxide Membranes

Bo Fang[a,b,*], *Zeyu Zhao*[a,c], *Jie Ma*[a,d], *Heng Li*[a,d,*], *Feng Yan*[a,c,*] *Xiaoming Tao*[a,b,*]

[a]Research Institute for Intelligent Wearable Systems, The Hong Kong Polytechnic University, Hong Kong, 999077 China.

[b]School of Fashion and Textiles, The Hong Kong Polytechnic University, Hong Kong, 999077 China.

[c]Department of Applied Physics, The Hong Kong Polytechnic University, Hong Kong, 999077 China.

[d]Department of Building and Real Estate, The Hong Kong Polytechnic University, Hong Kong, 999077 China.

* Address correspondence to bofang@polyu.edu.hk; heng.li@polyu.edu.hk; feng.yan@polyu.edu.hk; xiao-ming.tao@polyu.edu.hk

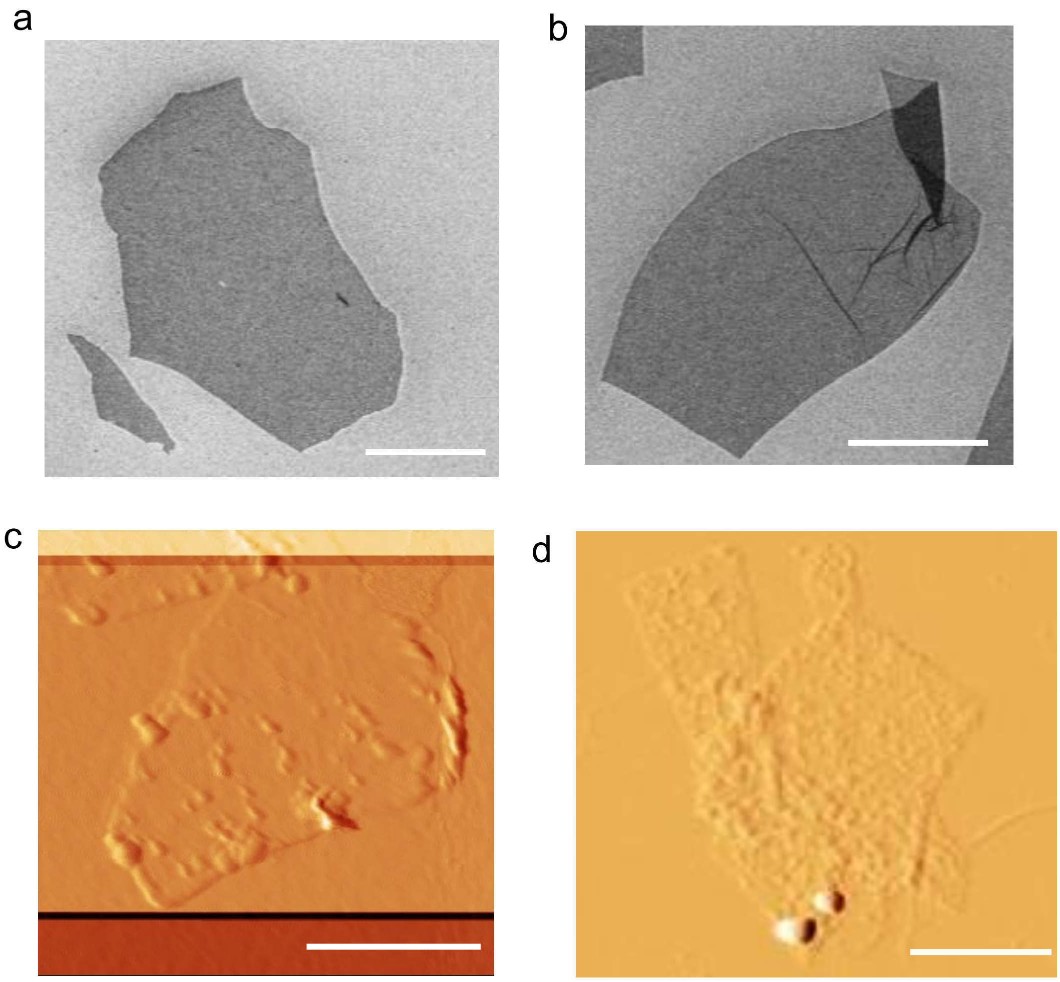

Figure S1. Scanning electron microscope (SEM) and atomic force microscope (AFM) images of graphene oxide (GO, a, c) and graphene oxide- polyethyleneimine (GO-PEI, b, d) sheets. The AFM observation of all the samples were conducted by diluting GO or GO-PEI samples at a very low concentrations, and the whole process were conducted at temperatures below 20 °C. Scale bars in a-d: 1 μm.

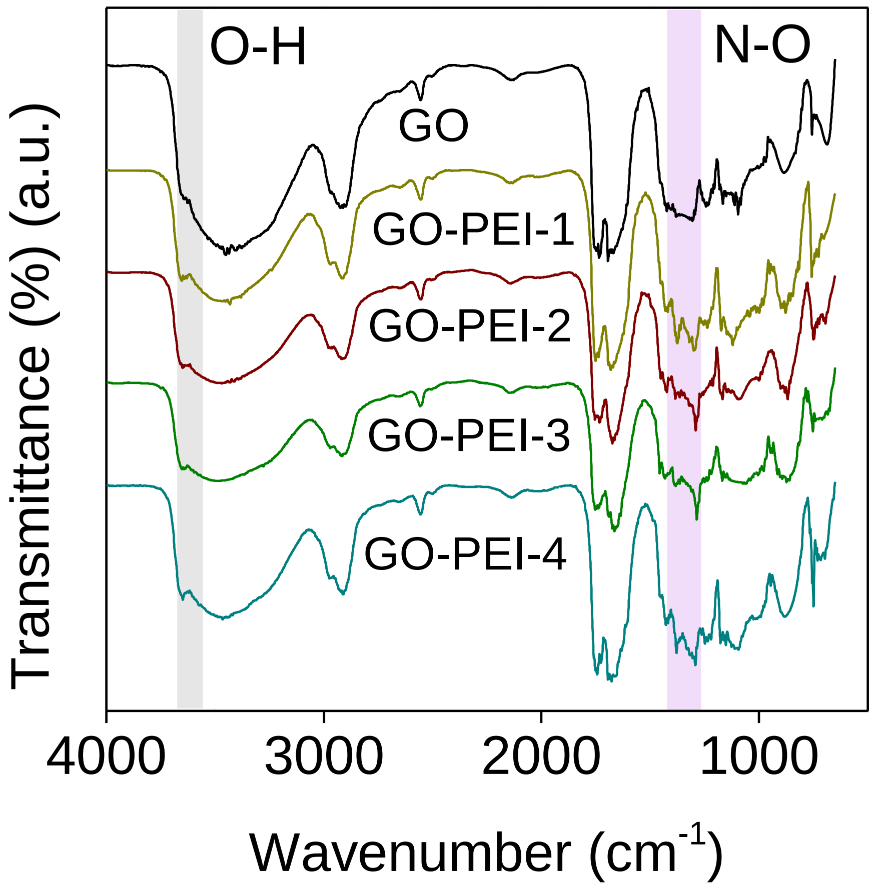

Figure S2. Fourier transform infrared (FTIR) characterization to the GO-PEI interactions. FTIR spectrum of all the GO and GO-PEI membranes.

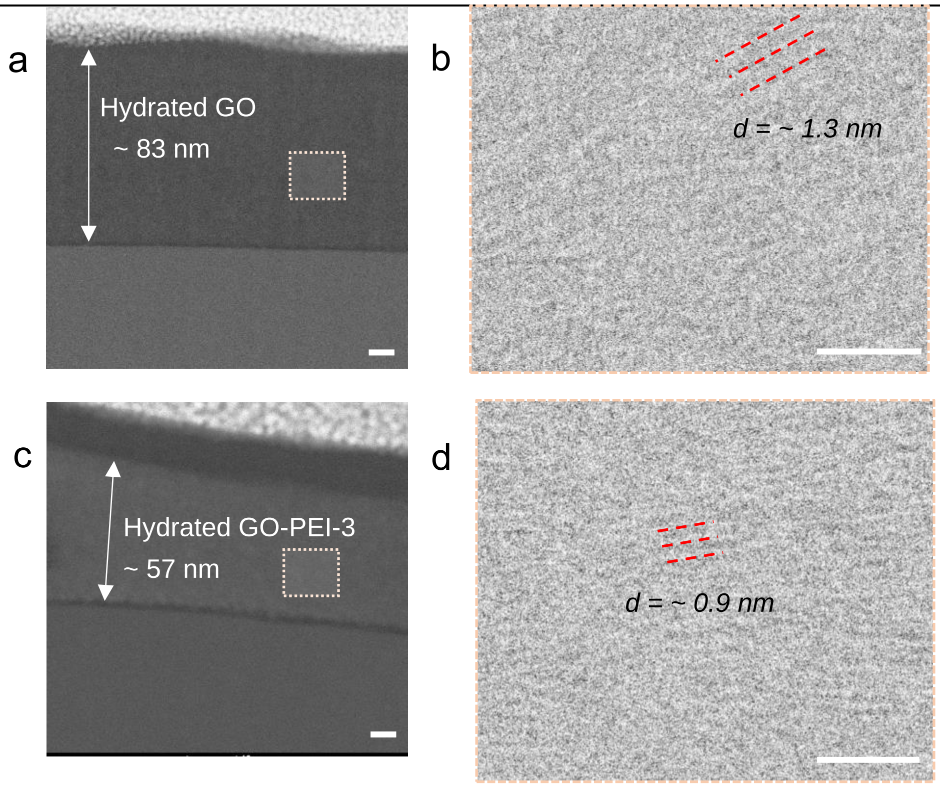

Figure S3. High-resolution transmission electron microscopy (TEM) observations of swollen membranes. TEM pictures (a) and enlarged TEM observation (b) showed the evolution of hydrated GO membranes. TEM pictures (c) and enlarged TEM observation (d) showed the evolution of hydrated GO-PEI-3 membranes. Scale bars: (a, c) 10 nm; (b, d) 5 nm.

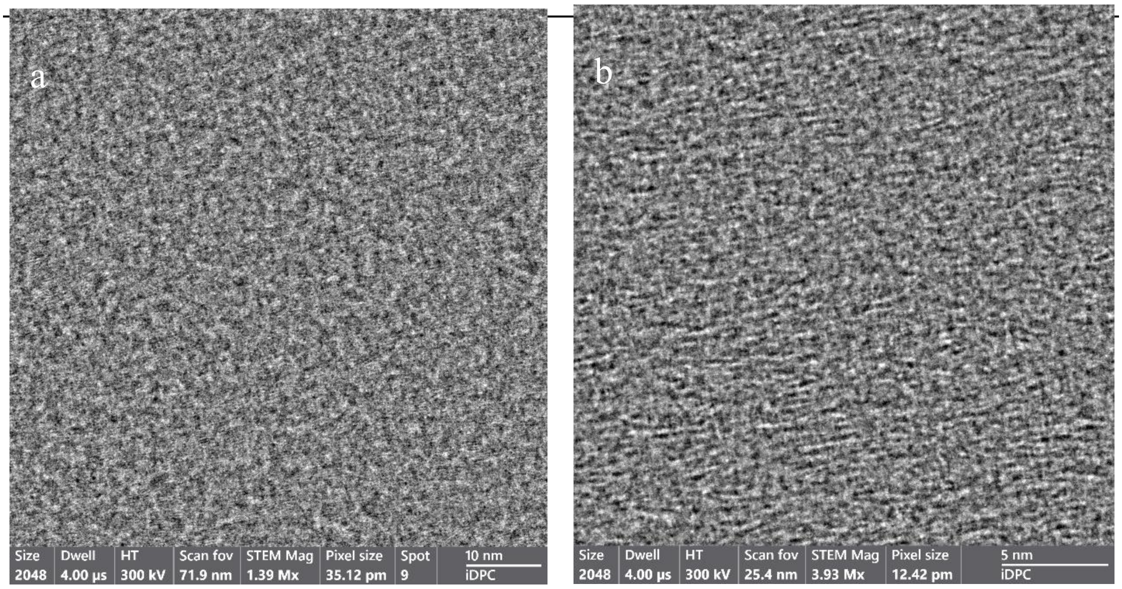

Figure S4. TEM observations of hydrated GO (a) and GO-PEI-3 (b) membrane. Based on the merging diffraction fringe, the stacking of GO sheets became more regular in GO-PEI-3 membrane than that of pristine hydrated GO membrane.

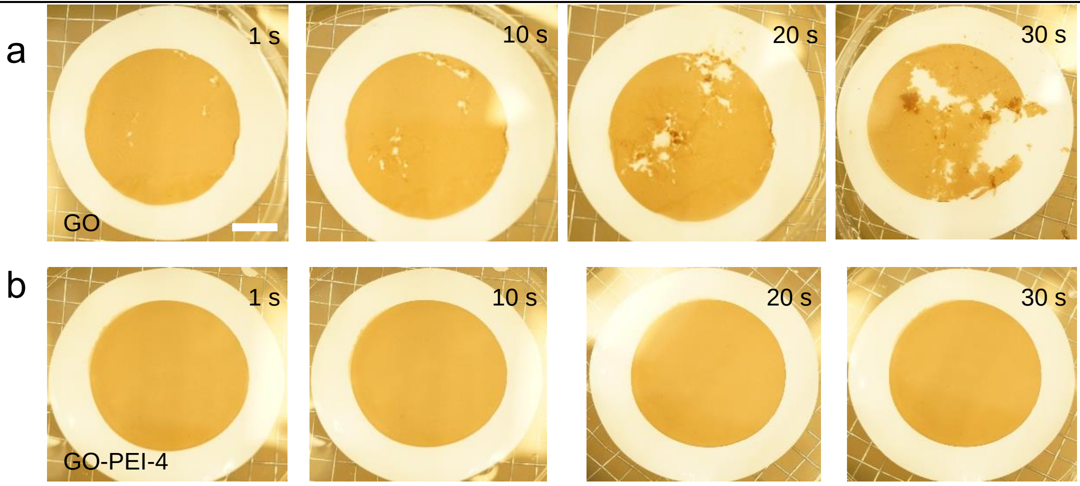

Figure S5. Ultrasonic test results of membranes. Ultrasonic test of GO membrane (a) and GO-PEI-4 membrane (b) during 30 seconds. Scale bar in a: 1 cm.

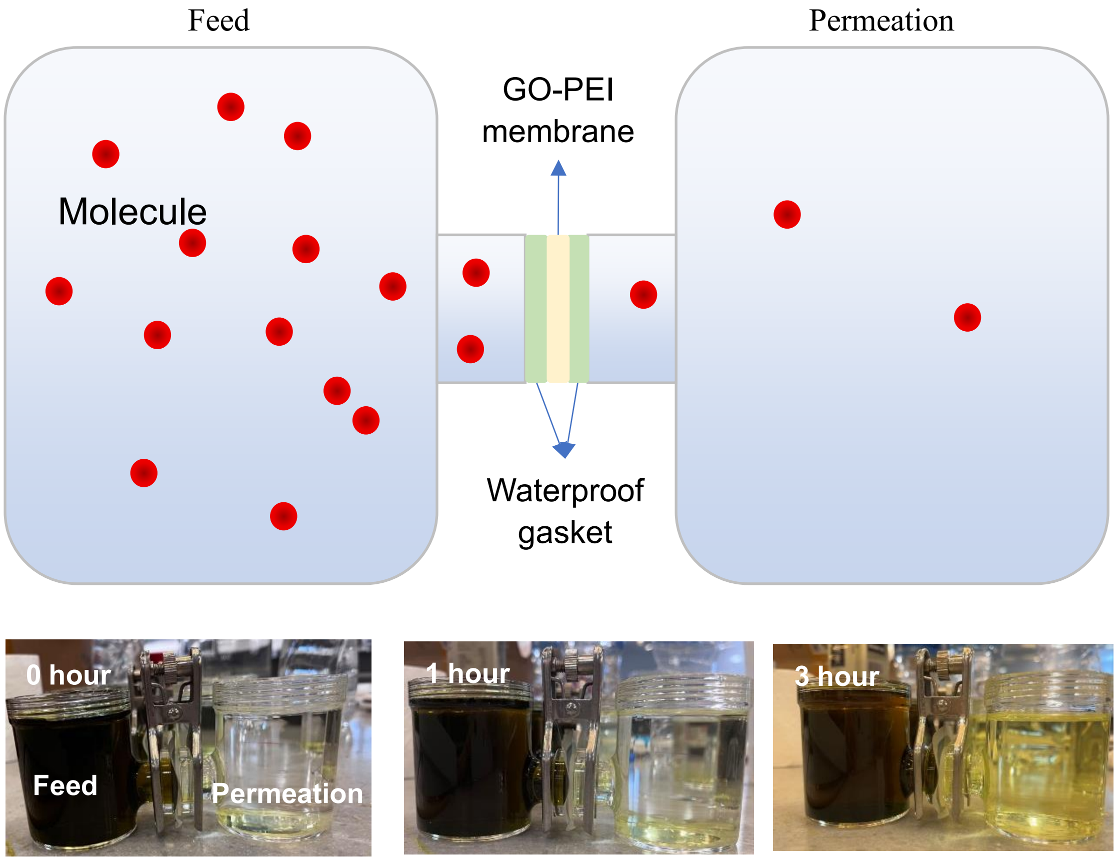

Figure S6. Schematic and real test of molecule permeation experiments. Molecule-sieving membranes were sealed between two glass contains with a volume of 100 ml. The pictures showed the filtration of $K_3[Fe(CN)_6]$ (feed 1 M) through GO membranes at 0 hour, 1 hour and 3 hour, respectively. The color of permeation side gradually became yellow with the filtration of $[Fe(CN)_6]^{3-}$ ions.

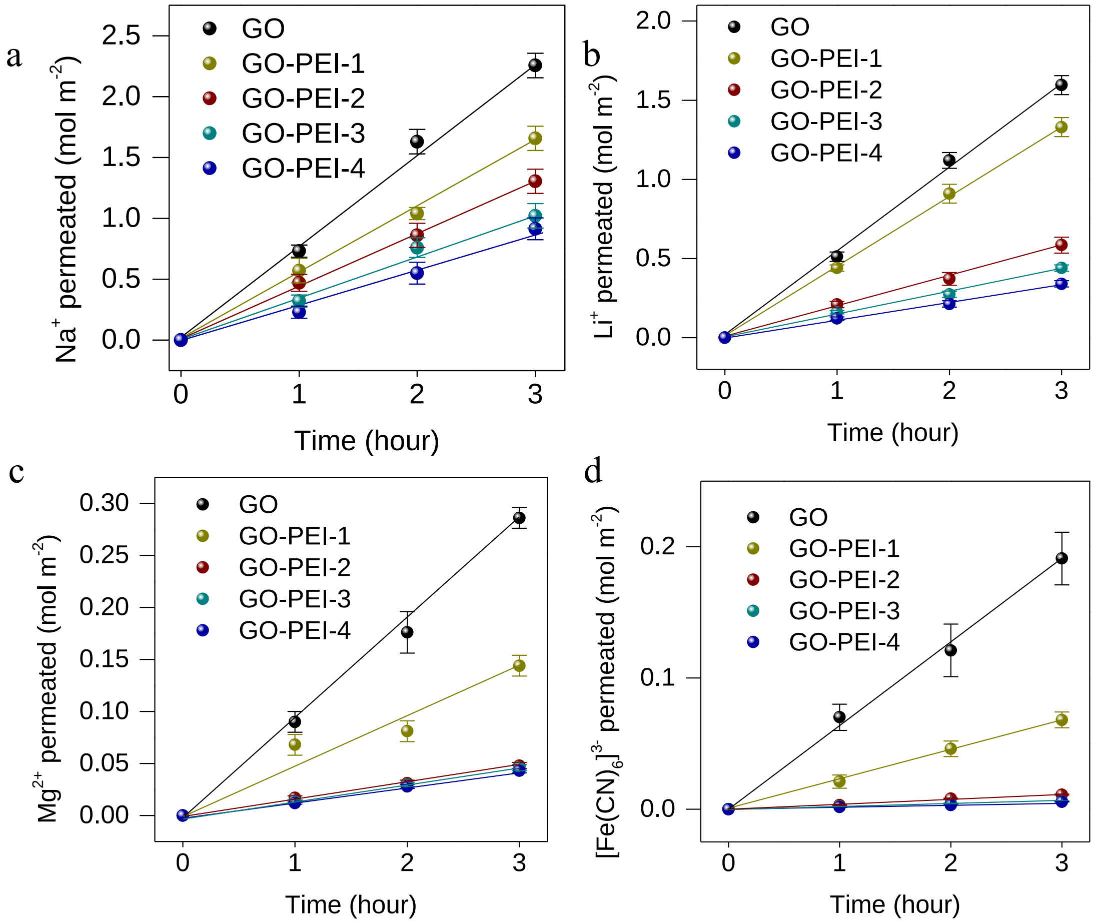

Figure S7. Permeation test results of ions through membranes. After 3 hours permeation, Na^+ (a), Li^+ (b), Mg^{2+} (c), and $[Fe(CN)_6]^{3-}$ (d) exhibited the decreasing permeation rates from ~ 2 mol m^{-2} to ~ 0.2 mol m^{-2}.

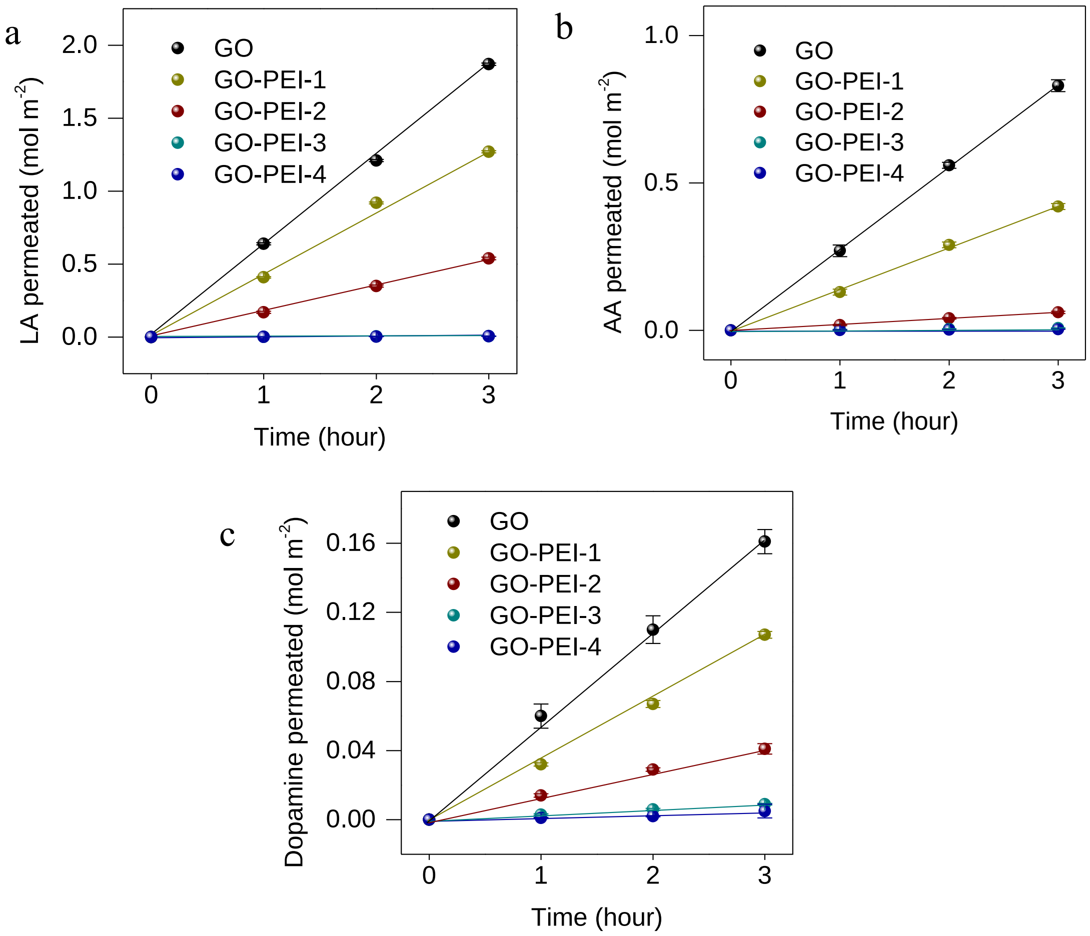

Figure S8. Permeation test results of biomarkers through membranes. After 3 hours permeation, lactate (LA, a), ascorbic acid (AA, b), and dopamine (c) showed the declining permeation rates from ~ 2 mol m^{-2} to ~ 0.16 mol m^{-2}.

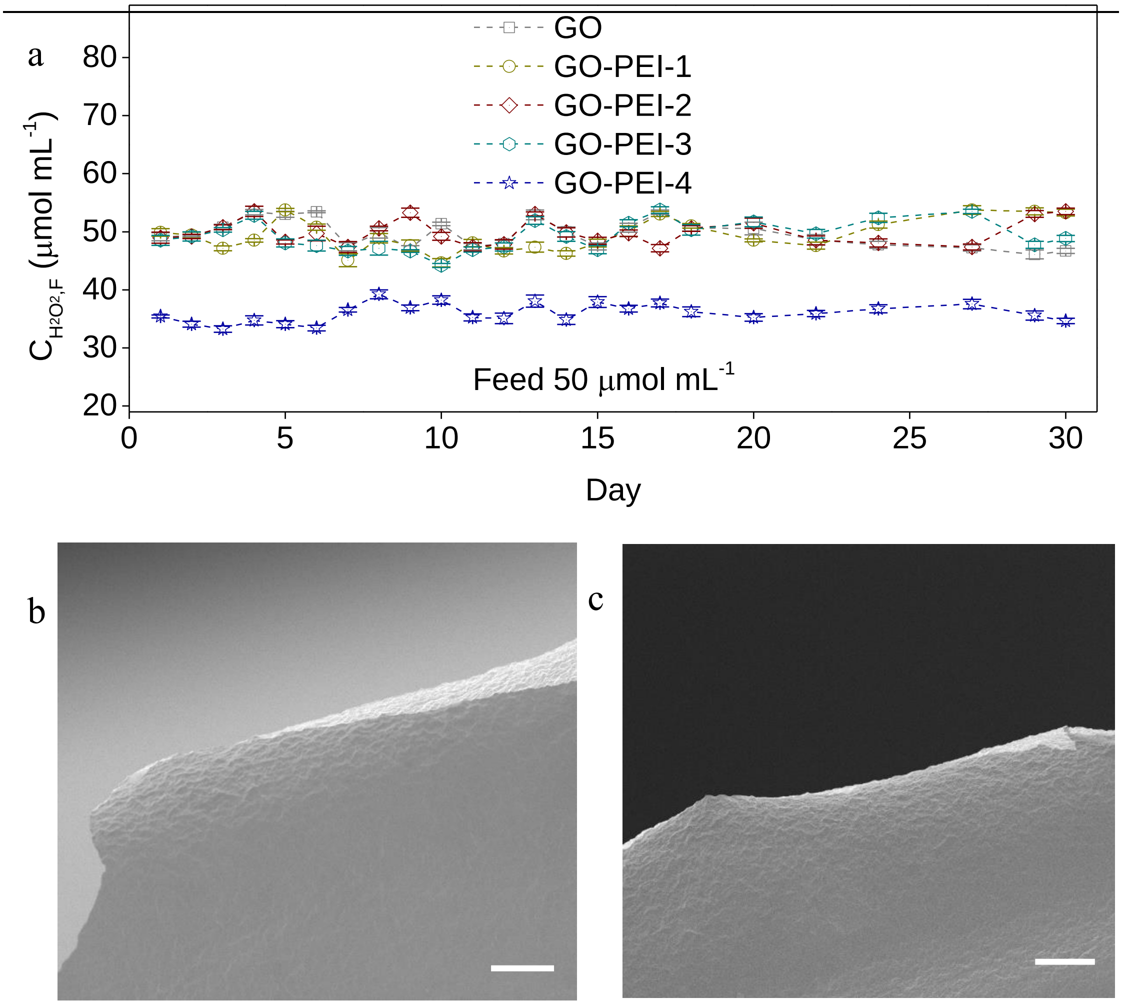

Figure S9. 30 days permeation test results of H_2O_2 through membranes. When feeding 50 μmol mL^{-1} H_2O_2 solutions, the H_2O_2 concentrations of collected filtrate ($C_{H2O2,F}$) through all the membranes. During the tests, the GO-PEI-4 membranes kept reliable structural stability, judging from the scanning electron microscope (SEM) observation of membranes at the first day (b) and the thirtieth day (c).

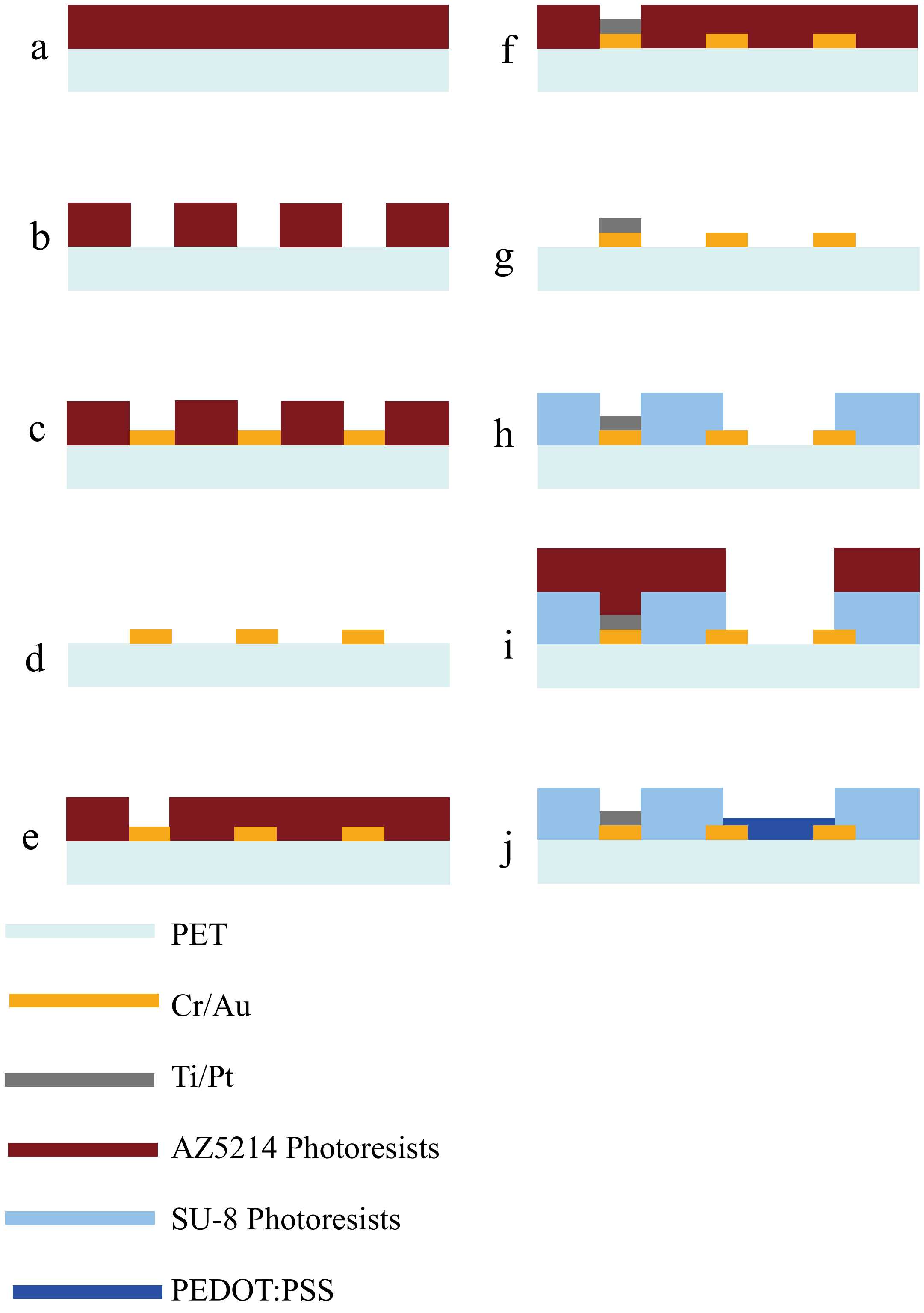

Figure S10. Fabrication process of organic electrochemical transistor (OECT). The details are explained at Methods. In this illustration, (a-d) Cr/Au electrode deposition. (e-g) Ti/Pt electrode deposition. (h) SU-8 photoresists encapsulation. (i-j) Patterning of PEDOT:PSS channel semiconductor.

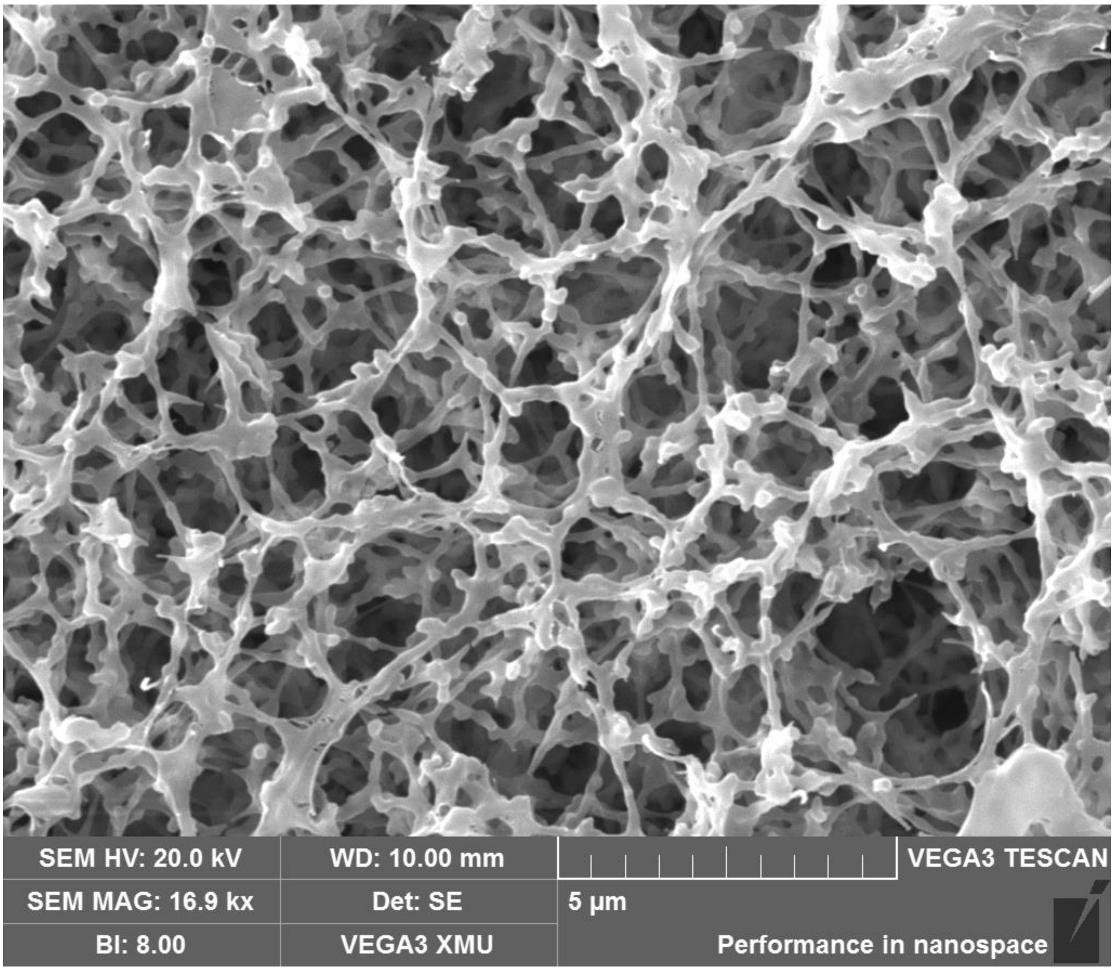

Figure S11. Microstructures of polymer substrates. Uniform pores with average sizes beyond 2 micrometers distribute uniformly in the polymer substrates.

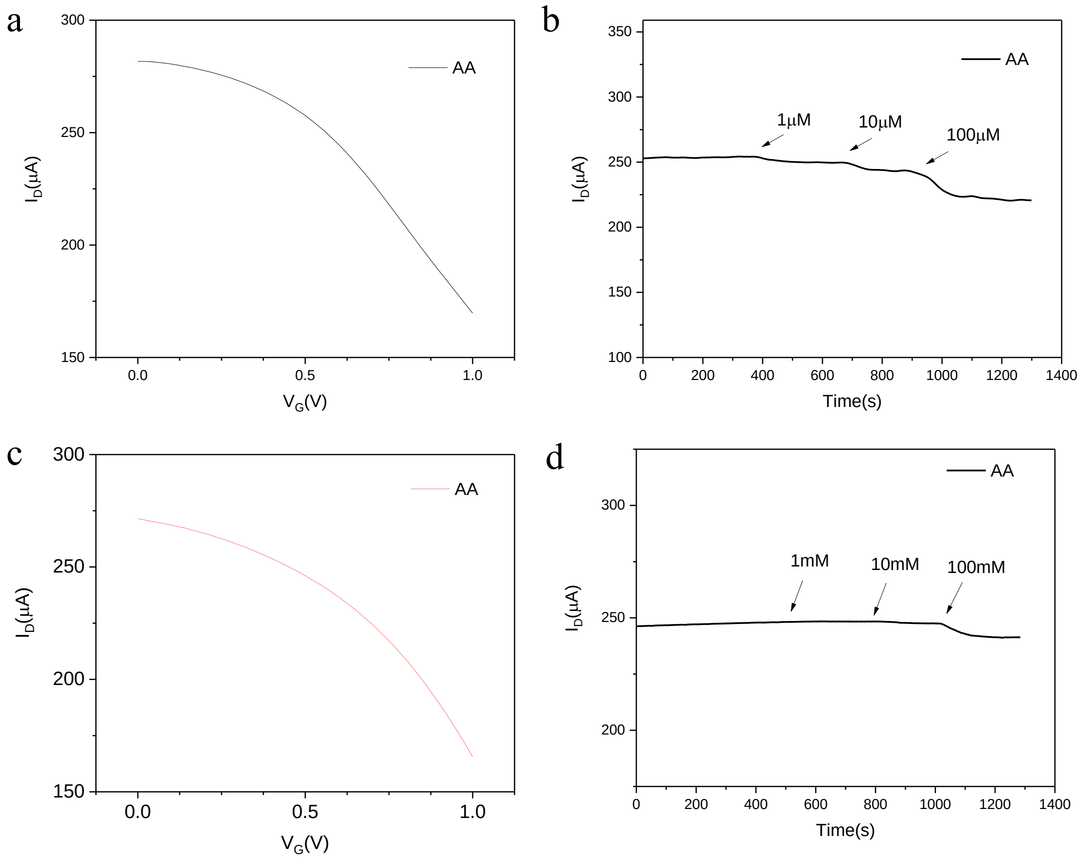

Figure S12. Transfer curves and Time-I_D tests of AA response on gate electrodes without membranes (a-b) and with GO-PEI-3 membranes (c-d). On bare electrodes, OECT respond to AA at a very low concentration of 1 μM, which improved to 100 mM when coating with GO-PEI-3 membranes.

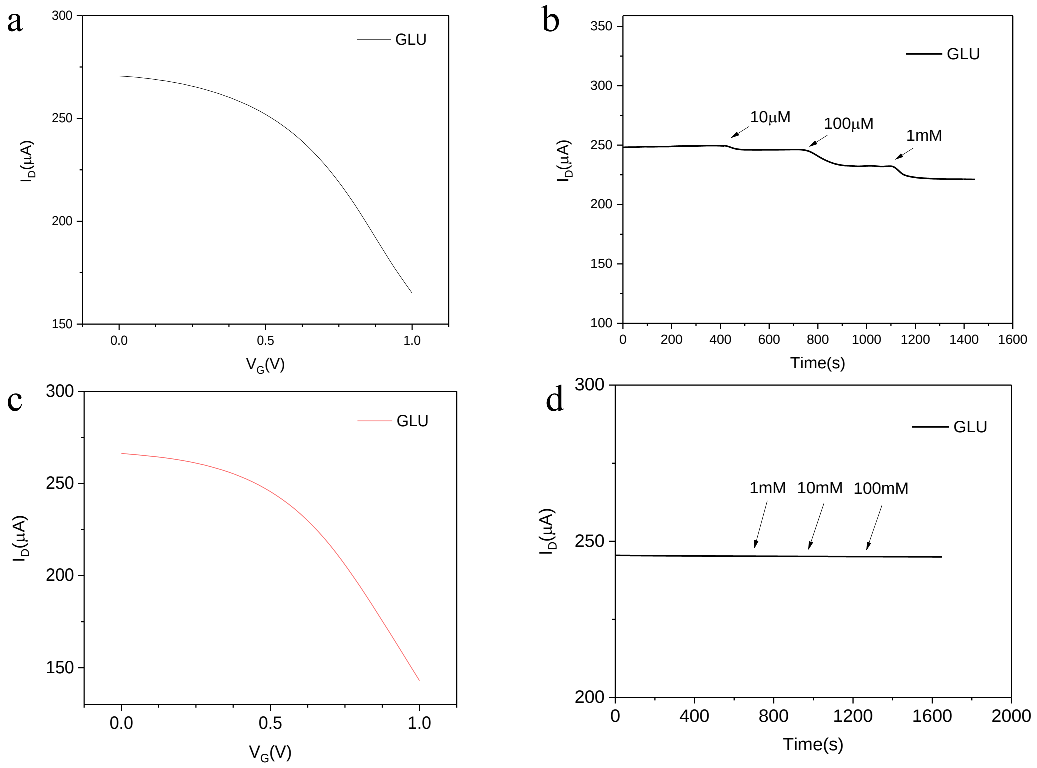

Figure S13. Transfer curves and Time-I_D tests of glucose (GLU) response on gate electrodes without membranes (a-b) and with GO-PEI-3 membranes (c-d). Loading with lactate oxidase (LOx), OECT respond to AA at a very low concentration of 10 μM on bare electrodes, which improved to > 100 mM when coating with GO-PEI-3 membranes.

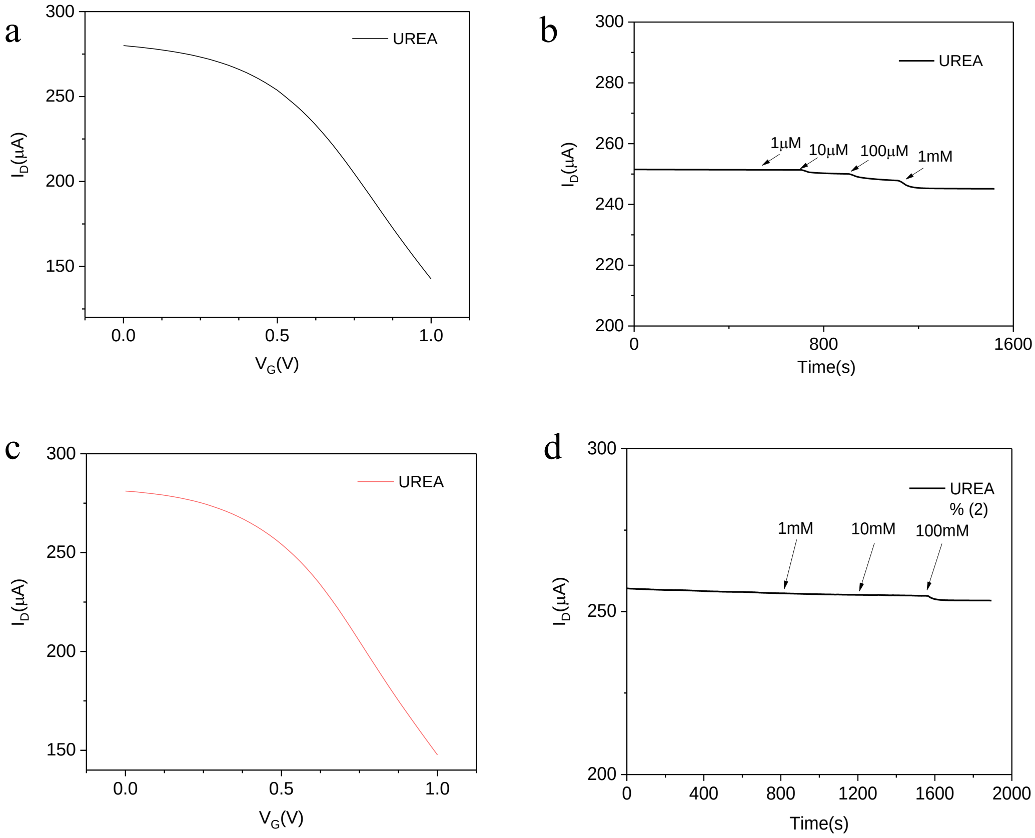

Figure S14. Transfer curves and Time-I_D tests of urea (UREA) response on gate electrodes without membranes (a-b) and with GO-PEI-3 membranes (c-d). On bare electrodes, OECT respond to AA at a very low concentration of 1 μM, which improved to > 10 mM when coating with GO-PEI-3 membranes.

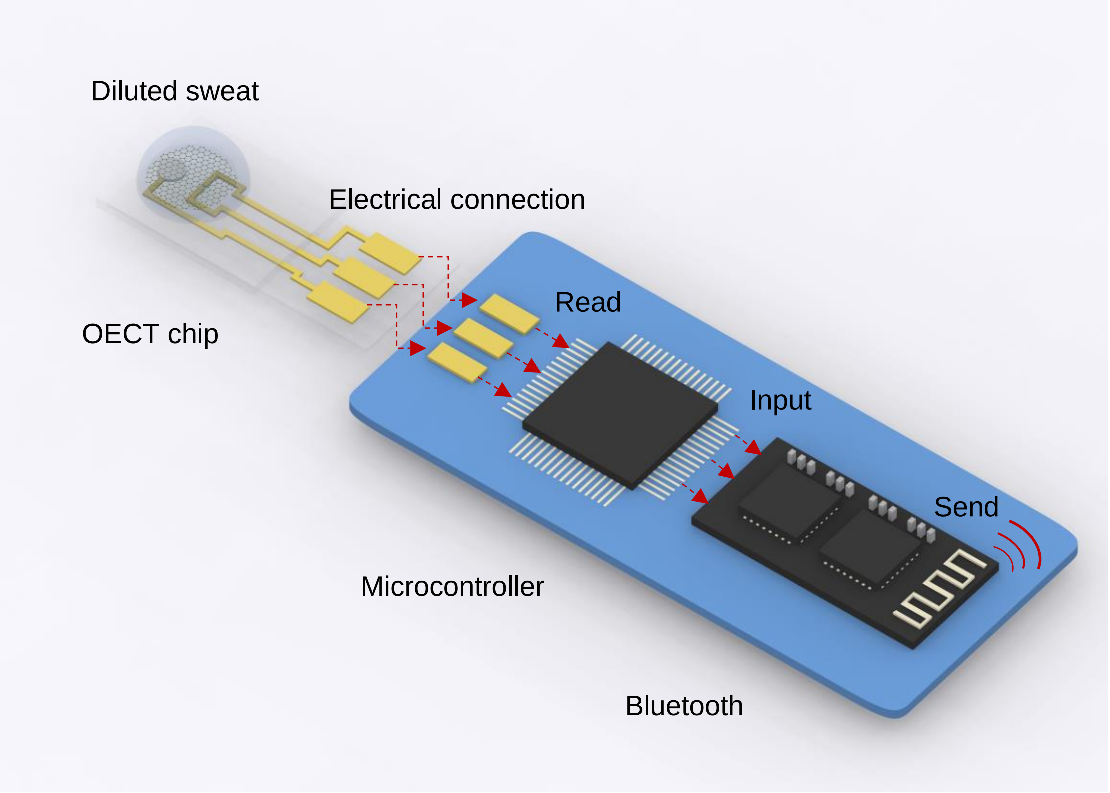

Figure S15. Operation flow of OECT chip. The real-time concentration of sweat lactate was sent to mobile devices via microcontroller and Bluetooth.

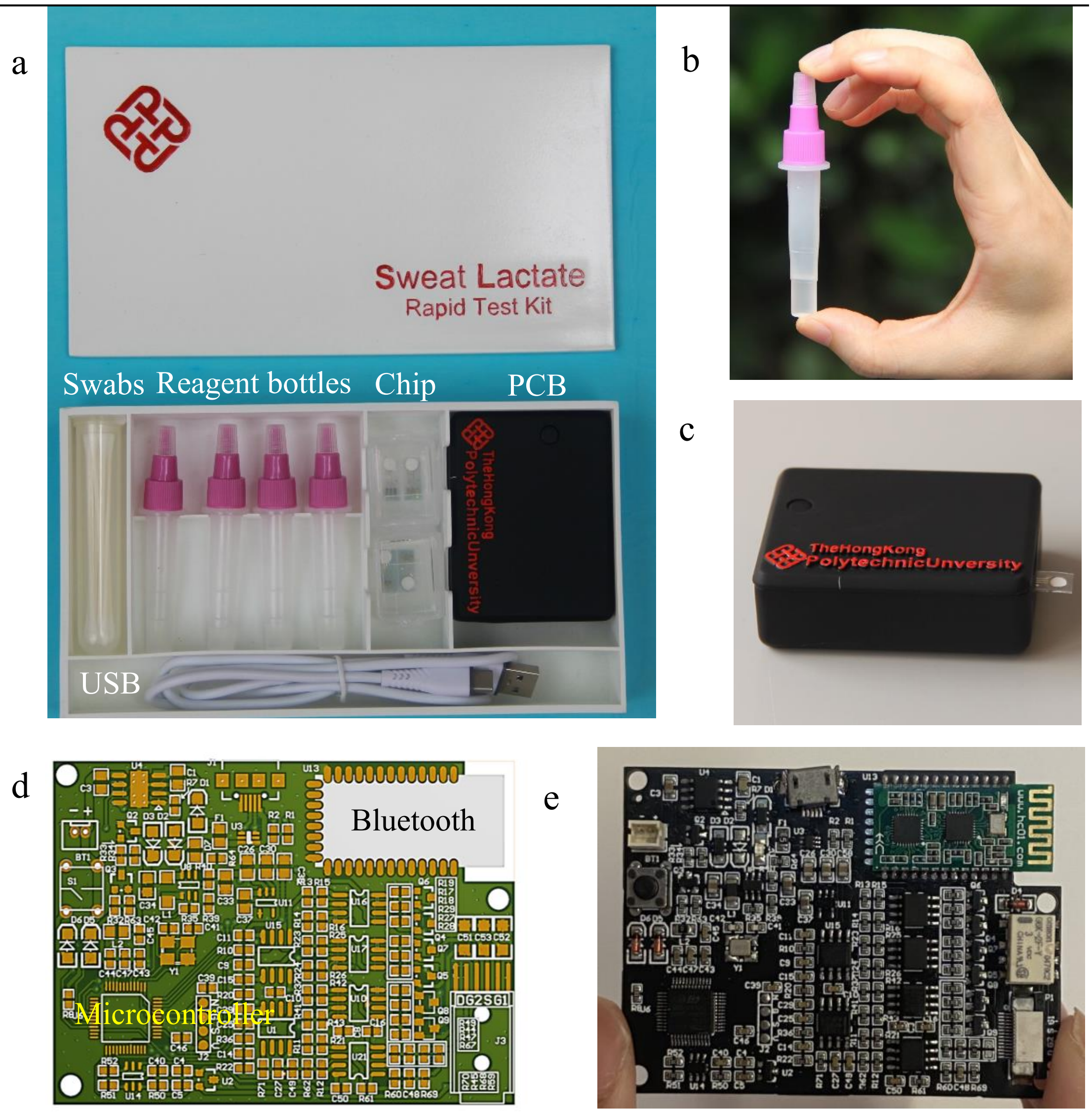

Figure S16. Details of the sweat lactate rapid test kit. When using the kit (a) for the rapid test of sweat lactate, we collected sweat with swabs, and then diluting and reserving sweat samples in the reagent bottles (b), further reading the concentrations of lactate by adding one drop of sweat samples on the OECT chip connected at PCB (c). Supplementary Fig. 10d-c shows the circuit diagram and picture of the PCB, respectively, including a microcontroller to read data and Bluetooth to send data to mobile devices.

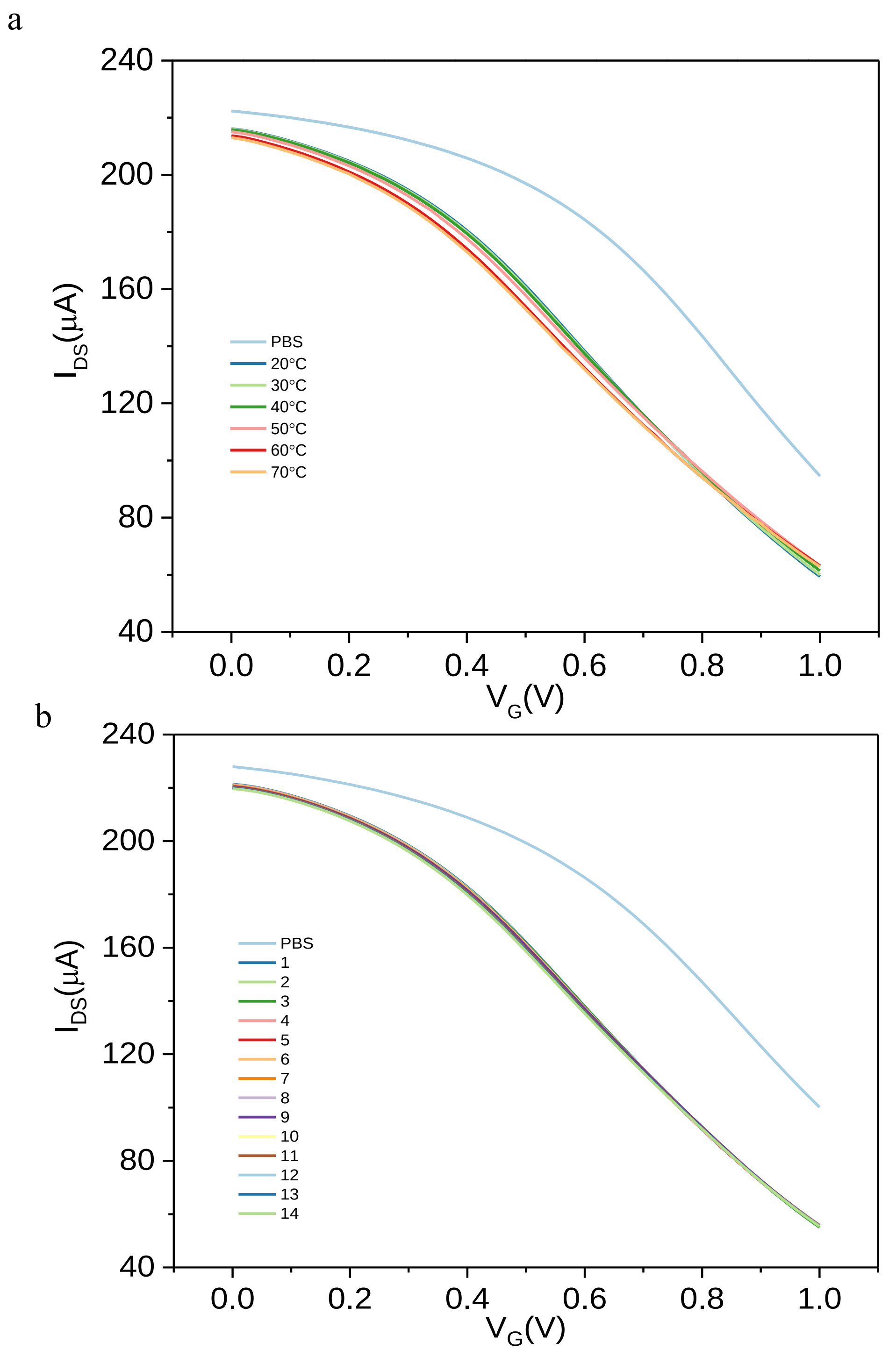

Figure S17. Study on the impacts of surrounding temperatures at transfer curves of OECT chip from 20 to 70 °C (a) and the durability test at constant 30 °C (b). High temperatures could influence on the decomposition rate of H_2O_2, producing negative effects on the stability of our OECT chip and sweat lactate rapid test kit. Based on tests between 20 and 70 °C, we found that the OECT chip had stable transfer curves between 20 and 40 °C. The almost unchanged transfer curves at 30 °C demonstrated the operation stability at near room temperatures.

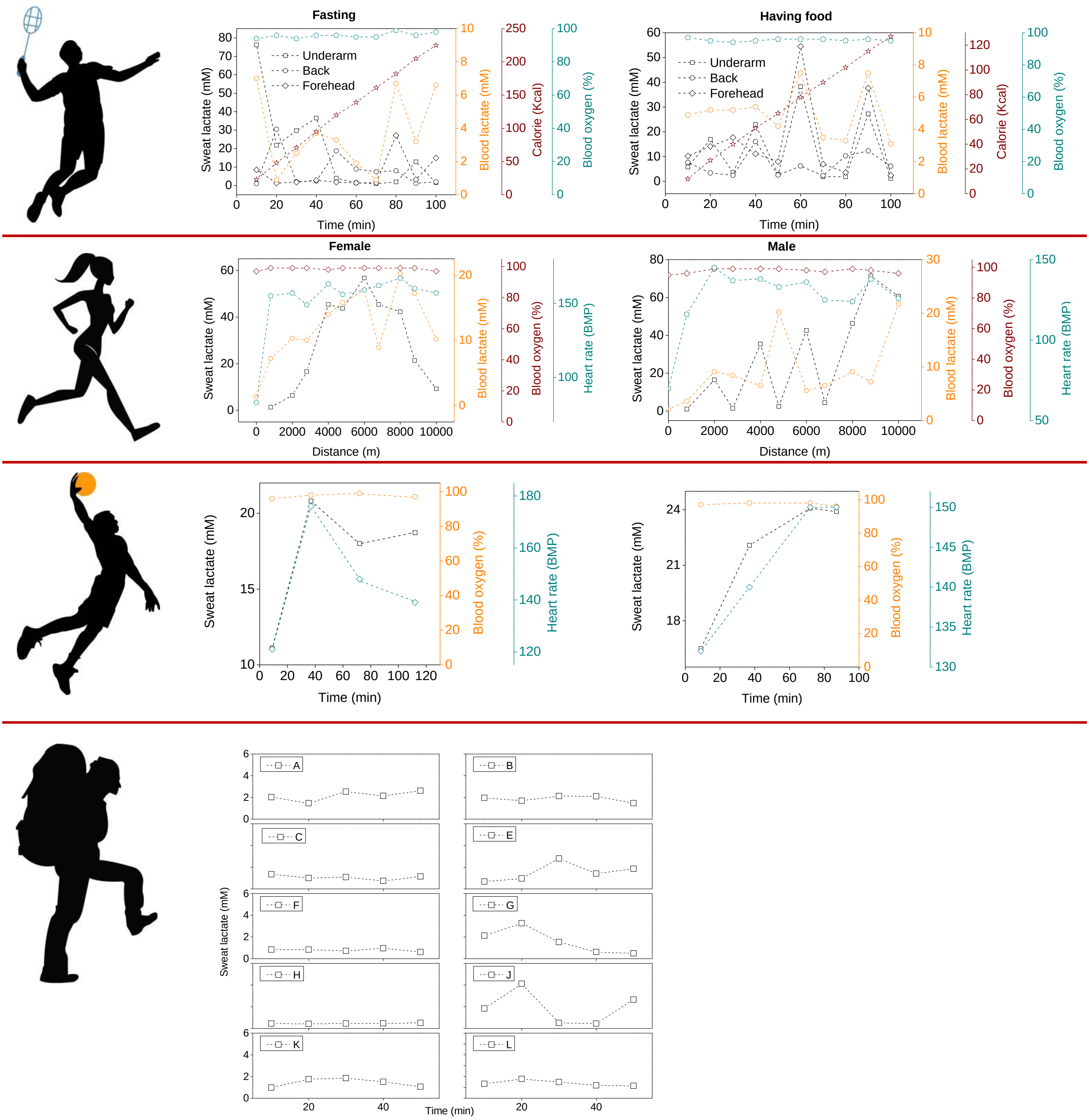

Figure S18. Real-time test to human participants during the continuous badminton, running, basketball and hiking exercise. Taking badminton exercise for an example, the concentrations of sweat lactate increased gradually at a fluctuant mode, a reaction of human circulatory system handling the excessive lactate in body fluids[1]. According to our tests, sweat lactate will be approaching to 20 mM at fatigue state, ~ 20 minutes

during playing badminton and ~ 2000 metre during running, while exceeding 35 mM when feeling high fatigue, i.e., ~ 60 minutes during playing badminton and ~ 4000 metre during running, respectively. The simultaneous measurements to other physiological signals, including blood lactate, calorie consumption, blood oxygen and hear rate, reflected the positive correlation between sweat lactate and blood lactate. This is because partial lactate in sweat is transported from blood[2].

References

1. Adeva-Andany, M. et al. Comprehensive review on lactate metabolism in human health. *Mitochondrion* **17**, 76-100 (2014).

2. Brooks, G. A. et al. Lactate in contemporary biology: A phoenix risen. *J. Physiol.* **600**, 1229-1251 (2022).
